# ECG Sensor Card with Evolving RBP Algorithms for Human Verification

**DOI:** 10.3390/s150820730

**Published:** 2015-08-21

**Authors:** Kuo-Kun Tseng, Huang-Nan Huang, Fufu Zeng, Shu-Yi Tu

**Affiliations:** 1 Department of Computer Science and Technology, Harbin Institute of Technology, Shenzhen Graduate School, Shenzhen 518055, China; E-Mail: 12S051058@hitsz.edu.cn; 2 Department of Mathematics, Tunghai University, Taichung 40704, Taiwan; 3 Department of Mathematics, University of Michigan, Flint 48502, MI, USA; E-Mail: sytu@umflint.edu

**Keywords:** electrocardiogram verification, biometric, access control system, non-stationary, wavelet, ECG complex, MIT-BIH database

## Abstract

It is known that cardiac and respiratory rhythms in electrocardiograms (ECGs) are highly nonlinear and non-stationary. As a result, most traditional time-domain algorithms are inadequate for characterizing the complex dynamics of the ECG. This paper proposes a new ECG sensor card and a statistical-based ECG algorithm, with the aid of a reduced binary pattern (RBP), with the aim of achieving faster ECG human identity recognition with high accuracy. The proposed algorithm has one advantage that previous ECG algorithms lack—the waveform complex information and de-noising preprocessing can be bypassed; therefore, it is more suitable for non-stationary ECG signals. Experimental results tested on two public ECG databases (MIT-BIH) from MIT University confirm that the proposed scheme is feasible with excellent accuracy, low complexity, and speedy processing. To be more specific, the advanced RBP algorithm achieves high accuracy in human identity recognition and is executed at least nine times faster than previous algorithms. Moreover, based on the test results from a long-term ECG database, the evolving RBP algorithm also demonstrates superior capability in handling long-term and non-stationary ECG signals.

## 1. Introduction

Access control systems using fingerprint, face, and iris biometric authentications [1] are extensively applied, but some issues, such as privacy and cost, still need to be addressed. Recently, security-related studies have shown a tendency to focus on realistic electrocardiogram (ECG or EKG) identifications/verifications for access control.

An ECG is a voltage variation signal that detects the electrical changes of the heart on the skin which are caused when the heart muscle depolarizes during each heartbeat.

The purpose of this paper is to propose a statistical-based algorithm using the reduced binary pattern (RBP) for human identity recognition. The suggested algorithm meets the accuracy and cost requirements in an ECG verification system. Any ECG signal will first be converted into concise binary patterns and then statistical counting and ranking processes follow for verification purposes. Its advantages will be illustrated as follows:

- Signal preprocessing, such as de-noising, adjusting signal means, and so on, can be totally neglected. The noise can simply be viewed as one verification feature.
- Unlike other published methods, ECG QRS (Quantitative Regression Swedish) detection can be waived completely in our scheme. The algorithm can be performed directly and still maintains robustness to dynamic variation of ECG signals.
- Variations in length and sampling rates of matching signals are absolutely allowed.
- The algorithm requires less ECG information content and performs in a timely manner with low computational complexity. It does not need ECG information content like R-R intervals, mean and variation of ECG signals, and so on.

The remaining parts of the paper are organized as follows. An overview of related works on ECG verification is presented in Section 2; the outline of the proposed algorithm is introduced in Section 3, followed by a detailed description; the experimental results are shown and discussed in Section IV; and some concluding remarks are drawn in Section V.

## 2. Related Studies

**Figure 1:**
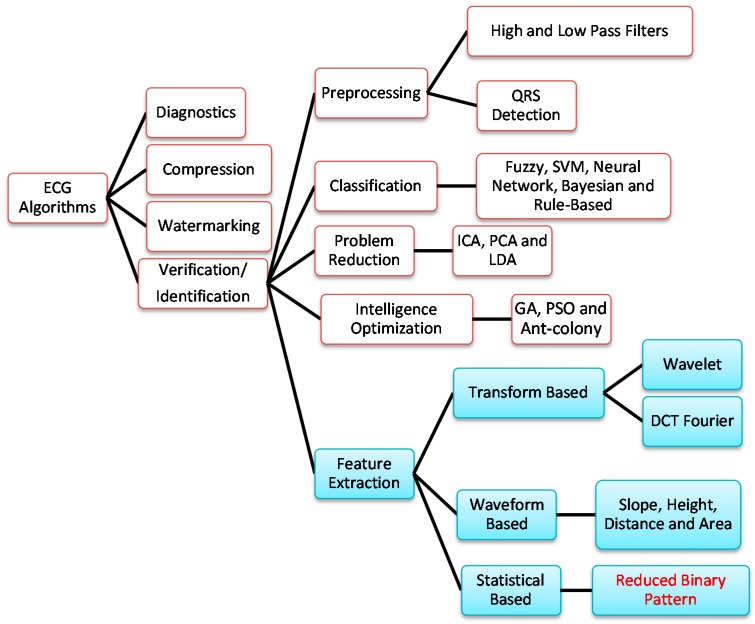
The classification of ECG algorithms.

The classification of ECG algorithms is shown in Figure 1. In this figure, FFT and DCT denote the fast Fourier transform and discrete cosine transform, respectively; SVM denotes the support vector machine; ICA, PCA, and LDA denote independent component analysis, principal component analysis, and linear discriminant analysis, respectively; GA and PSO denote genetic algorithm and particle swarm optimization, respectively. In addition to ECG-based diagnostics for heart diseases [2,3], ECG has also been applied to data compression [4,5,6], information watermarking [7,8], and human verification/identification [9,10,11,12,13,14,15,16,17,18,19,20,21,22,23].

Signal pre-processing, feature extraction, data classification, data reduction, and intelligence optimization are the key research focuses in human identification/verification. High- and low-pass filters and QRS detection are the main schemes employed in signal preprocessing, while fuzzy rule [24], SVM [13], neural network [2], Bayesian [25], and rule-based [26] algorithms are frequently adopted in data classification. For data reduction, ICA [27], PCA [17], and LDA [28] have been used in ECG signal processing. Intelligence optimization techniques such as GA [29], PSO, ant-colony, and so on are commonly utilized for tuning the parameters of the aforementioned algorithms.

Most ECG-based human identification/verification methods rely on feature extraction derived from the ECG signals. The features are usually extracted according to three models: transform-based, waveform-based, and statistical-based.

The transform-based algorithms consist of wavelet transforms [9,10,11,12,13,14] and frequency domain transforms, including Fourier transform [15] or DCT [16]. Since the wavelet transform contains information in the time and frequency domain, it is more popular than the frequency domain transform.

Correlation coefficients and measurements of wavelet distances have been used in matching acquired ECG signals and modules [9]. Since this identification/verification method requires heavy calculation, its implementation is restricted in the statistical sense. The feature selection algorithm in [12] applied the feature set evaluation (FSE) k-nearest neighbor (k-NN) algorithm to improve low recognition rates and used the eigenspace method to reduce data dimensions; however, this approach is both complicated and time-consuming. In [13], morphological characteristics are first extracted through the wavelet transform and the independent component analysis; SVM follows for identification/verification purposes. Although a high identification rate could be reached, a lengthy feature extraction process seems unavoidable.

Waveform-based algorithms [17,18,19,20,21,22,23] extract different time domain characteristics (distance, height, and area) from fiducial points inside the ECG waveform. These waveform descriptors will be used to match or classify ECG signals in the identification/verification process. These algorithms usually have good accuracy in recognizing regular ECG signals but show opposite results for irregular data.

Some researchers combined a precision-matched result with a waveform neural network in the signal preprocessing stage [18]. This model extracted seven features from the ECG signals based on their amplitude and the interval to be analyzed by the decision-based neural network. The computational complexity depends heavily on the forms of those time-domain ECG signals and the level of difficulty of the matching process carried out by the neural network. Nineteen characteristics are extracted from the time interval, amplitude, and angle of deflection and studied [22]; the identification is examined using Euclidian distances and an adaptive threshold. The eigenvectors used in feature-matching take time but are necessary for all band waves in the ECG signals.

An ECG signal can be described as a non-stationary time series that presents some irregularities in the waveform. Unlike the waveform-based algorithms, the transform-based algorithms analyze the non-stationary information based on the signal’s presentation in the frequency domain. Not only is this process slow, but it is also difficult to extract good features for the purpose of identification.

Statistical-based algorithms usually depend on statistical evaluations (count, mean, and variance) of human identification. They are usually less time-consuming but definitely need a well-designed statistical model to assure high-quality accuracy. A method based on rank order statistics was presented to analyze the human heart beat [30].

The non-stationary behavior of ECG has been utilized in many studies. The fetal ECG was reconstructed with higher-order statistical tools exploiting ECG non-stationary properties associated with post-de-noising wavelets [27]. A de-trended fluctuation analysis to quantify the correlation property in the non-stationary physiological time series was presented [31]. Our previous work for an ECG card access control system [32] focused on the architecture in ECG human identification.

Compared with algorithms presented in the literature, our proposed scheme is capable of providing secure and accurate results with a user-possessed controller. Moreover, it can be easily embedded into the field application structure to ensure the implementation of a feasible ECG identification hardware.

## 3. System Architecture and Application Example

Even though some ECG biometric identifications have been demonstrated, there is a serious issue regarding the use of a centralized ECG database. Due to implementation cost and accuracy issues, there is not yet a feasible application. In our previous work [32], we put this idea into practice and introduced a portable ECG card for access control. This small ECG card provides a cheap and convenient way to enhance door access security.

**Figure 2:**
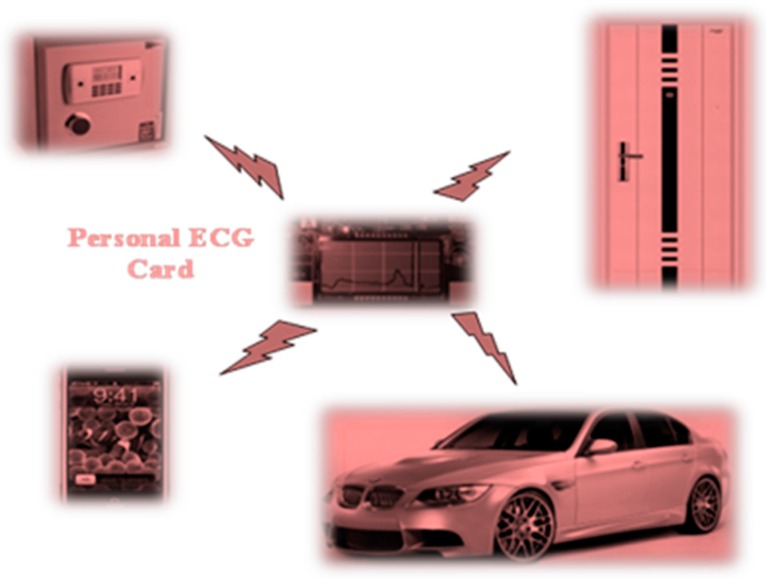
Application diagram of ECG sensor card for human verification.

An ECG access control system consists of a personal ECG sensor card and an access control device. An ECG card is a small device for storing personal ECG data and will be useful for identity recognition. As suggested in Figure 2, applications of ECG cards include secure personal keys to open cars, houses, deposit boxes, and mobile phones.

The following advantages mean that the ECG approach outperforms the most popular fingerprint approach.

1. The ECG signal is not visible and photo-copying is impossible. Replication of ECG signals is more difficult.
2. ECG data can be measured only by a smaller, low-power-consumption, low-cost, simple circuit.

**Figure 3:**
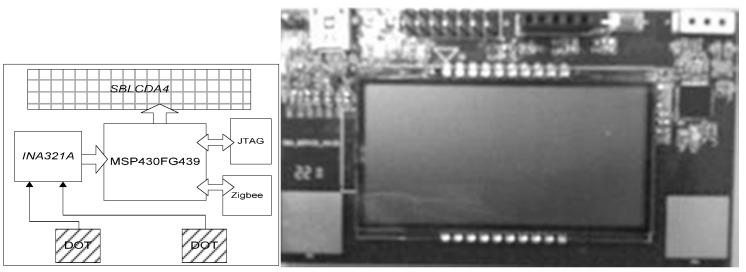
Architecture and hardware implementation of ECG sensor card.

The blueprint of the architecture of an ECG sensor card is shown on the left of Figure 3. Data are obtained through a contact pad (denoted by “DOT”), and the processing unit, the integrated chip “INA321A”, is in charge of common mode noise removal from the original signal. The main processing unit, the integrated microprocessor “MSP430FG439”, controls and transmits all data to the ZigBee module, a short-range wireless transmission module that communicates with the access control device. The other modules include “SBLCDA4” and “JTAG”, which will be used for LCD (Liquid Crystal Display) display and debugging the microprocessor, respectively.

One real implementation of the ECG sensor card is shown on the right of Figure 3. An ECG card contains two voltage-sensitive contacts, noise filter modules, a microprocessor, and a wireless transmission module. This small and low-cost device allows practical ECG identification in real life.

**Figure 4:**
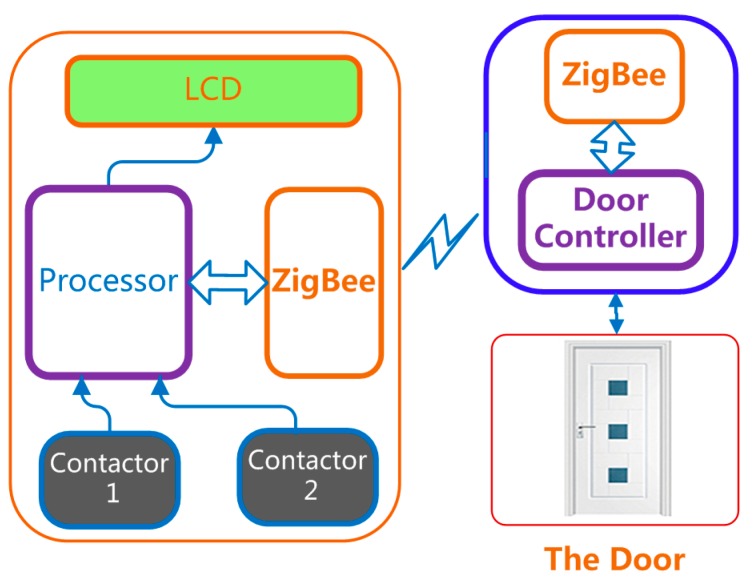
Door access control: Application of ECG sensor card.

**Figure 5:**
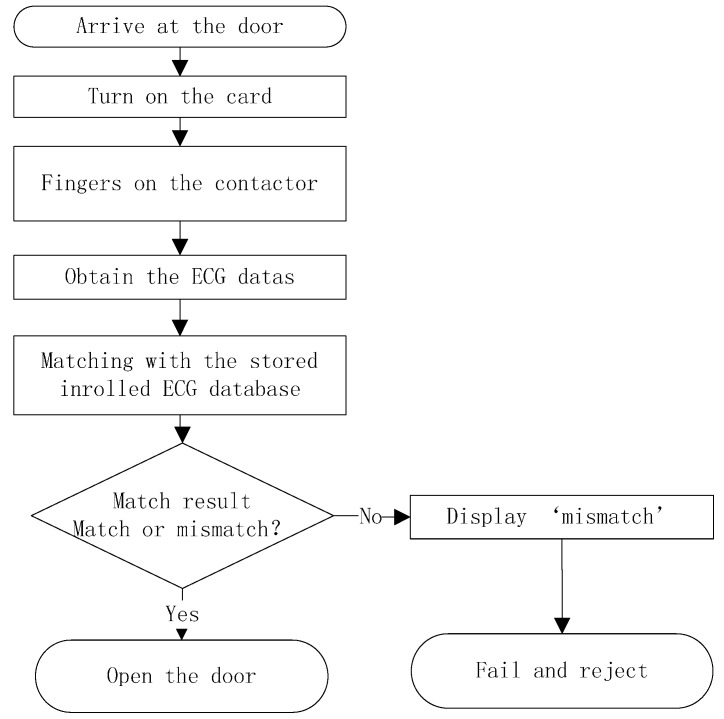
Flow chart of ECG verification processing.

A door access control, as shown in Figure 4, serves as one real application of ECG access cards. This card checks whether an ECG signal provided by the user matches that stored inside the card. The controller is an embedded system or personal computer connected with an ECG card via the wireless transmission module. The flowchart in Figure 5 shows the ECG verification process in the door access control.

## 4. Algorithm

### 4.1. The Basic RBP Algorithm

The idea of our proposed algorithm, RBP for ECG verification, is related to Yang’s [30] and Kumar’s [33] works, but we expand it to a different field of application. The differences between Yang’s model and our model are as follows:

1. Their approach focuses on the human heartbeats; ours focuses on just the bare ECG signals.
2. They convert, count, and rank P waves in the ECG signals only; we perform these procedures on every sample of ECG data to obtain the reduced binary pattern.
3. They aim for heart disease classification; we focus on human identity recognition through ECG signals.

The processing in our design can be roughly divided into three necessary steps that will be illustrated as follows.

**Step 1: Reduced Binary Pattern Conversion** 

All ECG signals are non-stationary. Consider an ECG signal as $\;x=\{x_{1},\;x_{2},\;x_{3},\ldots ,x_{N}\}$*,* where the real-valued $x_{i}$ corresponds to the $i^{\text{th}}$ input datum. Each pair of consecutive input signals is compared and the data are categorized into one of the two cases: a decrease or increase in $x_{i}$. A preliminary reduced function then maps these two cases to 0 or 1, respectively, according to the rule:

(1) $$y_{i}=\{\begin{array}{l} 0\;,\;x_{i+1}\;\leq \;x_{i}\\ 1\;,\;\;x_{i+1}\;>\;x_{i} \end{array}$$

This procedure converts the ECG signal of length N to a binary sequence $\;Y=\left\{y_{1},\;\;y_{2},\ldots ,y_{N-1}\right\}$ of length  N−1. Every  m  bits in Y are grouped to construct a reduced binary sequence of length  m, referred to as an *m*-bit word, and then all such words are collected to form a reduced binary pattern $B=\left\{b_{1},\;\;b_{2},\ldots ,b_{N-m}\right\}$ where $\;b_{k}=\left\{\;y_{k,\;}\;y_{k+1,\;}\ldots ,y_{k+m-1\;}\right\}$. We then convert each *m*-bit word $b_{k\;}$ to its decimal expansion $w_{k}$.

**Figure 6:**
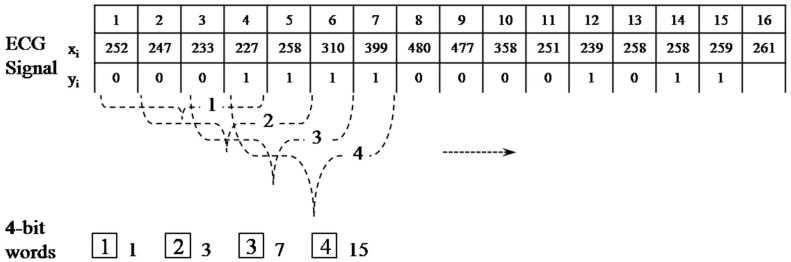
Process of the reduced binary pattern conversion.

An example of the reduced binary pattern conversion for  m=4  is depicted in Figure 6. For instance, the first four-bit word $b_{1}=\left\{0001\right\}\;$ is labeled as $w_{1}=1$, which equates to $\;0\times 2^{3}+0\times 2^{2}+0\times 2^{1}+1\times 2^{0}$.

**Step 2: Counting and Ranking Processes** 

**Figure 7:**
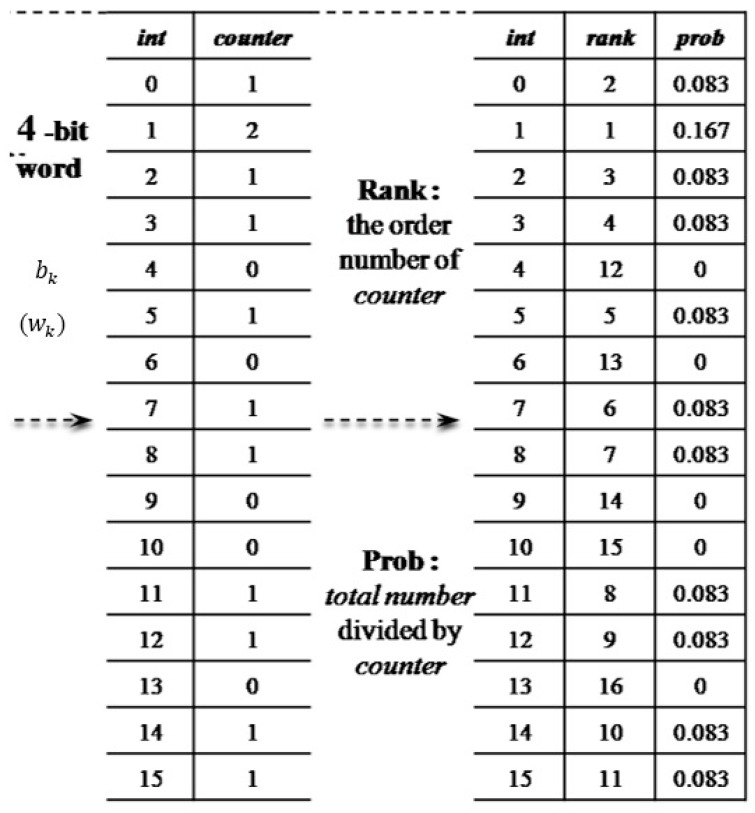
The counting and ranking process.

The main theme in this step, as shown in Figure 7, is to count the occurrences of all $\;w_{k}\;$ and sort them in order of descending frequency.

Let $w_{k}$ be an integer for k=1, 2, ⋯, N−m. It is obvious that values of $\;w_{k}$ range from 0 to $\;2^{m}-1$. Let integer $j\in w_{k}$, and let p(j) and $n_{j}$ be the corresponding relative frequency and occurrence of  j. To be exact, $p\left(j\right)=\frac{n_{j}}{N-m}\;$ and $\overset{\left(2^{m}-1\right)}{\underset{j=0}{\sum }}n_{j}=N-m$. Next, j  is ranked according to its frequency $n_{j}$ from the largest to the smallest. For example, R(j)=1 means the m-bit words $b_{k}$, which converts to the same j as those that appear the most frequently in the reduced binary pattern.

**Step 3: Measurement of Similarity** 

Consider two segments of ECG data $S_{1}$ and $S_{2}$, which may belong to two distinct subjects. To understand how closely they are related, the measurement of similarity needs to be defined. We incorporate a weighted distance formula [30] to measure the similarity between $S_{1}$ and $S_{2}$:

(2) $$d\left(S_{1},S_{2}\right)=\frac{\sum_{j=0}^{\left(2^{m}-1\right)}\;\left|R_{1}\left(j\right)-R_{2}\left(j\right)\right|\;p_{1}\left(j\right)\;p_{2}\left(j\right)}{\left(2^{m}-1\right)\cdot \sum_{j=0}^{\left(2^{m}-1\right)}p_{1}\left(j\right)p_{2}\left(j\right)}$$

where the segment means a sequence of sampled ECG data of 10 sample periods which serves as a basic unit for our analysis. Each sample period denotes the ECG signal in an R-R interval. p(j) and  R(j) represent the relative frequency and rank of  j  in the sequence ${\text{S}}_{i},\;i=1\text{ or }2$. The absolute difference between two ranks is multiplied by the normalized probabilities as a weighted sum; the factor $\;\frac{1}{2^{m}-1}\;$ assures that all values of measurements lie within the scope of (0, 1).

Consider two groups of ECG data, $S_{L}$ and $S_{K}$, each containing $\;m_{L}$ and $m_{K}$ segments, respectively. We define the measurement of similarity between these two groups:

(3) $$D\left(S_{L},S_{K}\right)=\frac{1}{m_{L}\cdot m_{K}}\underset{S_{1}\in S_{L}}{\sum }\underset{S_{2}\in S_{K}}{\sum }d\left(S_{1},S_{2}\right)$$

where $S_{1}$ and $S_{2}$ are the corresponding segments from $S_{L}$ and $S_{K}$, respectively; $d\left(S_{1},S_{2}\right)$ denotes the associated distance between these segments. $D\left(S_{L},S_{K}\right)$ is the average distance of all segments from $S_{L}$ and $S_{K}$. If $\;S_{L}=S_{K}$, $D\left(S_{L},S_{K}\right)$ is referred to as the intra-group distance; otherwise it is the inter-group distance.

### 4.2. The Advanced RBP Algorithm

Next, we consider a new scaling factor α, which is an increment in the length of the interval, in the RBP algorithm. All steps in this algorithm are similar to those performed in the basic design, except that the binary sequence will be obtained from the comparison of $x_{\left(\alpha \cdot i+1\right)}$ and $x_{\left(\alpha \cdot i-1\right)}$ instead of $x_{i+1}$ and $x_{i}$, where$\;x_{i}\;$represents the raw ECG data. The reduced function (1) is now replaced with:

(4) $$y_{\left(\alpha \cdot i-1\right)}=\{\begin{array}{l} 0\;,\;x_{\left(\alpha \cdot i+1\right)}\;\leq \;x_{\left(\alpha \cdot i-1\right)}\\ 1\;,\;x_{\left(\alpha \cdot i+1\right)}\;>\;x_{\left(\alpha \cdot i-1\right)} \end{array}$$

**Figure 8:**
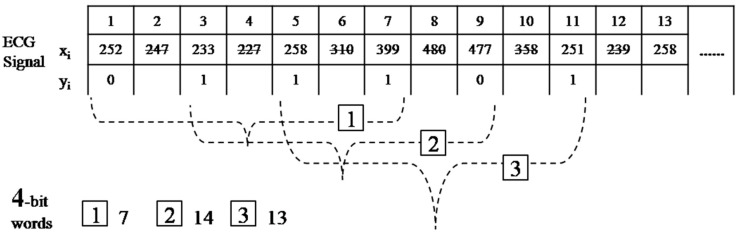
Process of the modified reduced binary pattern conversion.

Figure 8 represents the process of the modified RBP conversion of m=4  and α=2. The first four-bit word $b_{1}=\left\{0111\right\}\;$ is now labeled as $w_{1}=7=2^{2}+2^{1}+2^{0}\;$.

If we are concerned with the effect of the size of variations in amplitude, the reduced function will be:

(5) $$y_{i}=\{\begin{array}{c} 0\;,\;x_{i+1}\;\leq \;\beta +x_{i}\\ 1\;,\;\;x_{i+1}\;>\;\beta +x_{i} \end{array}$$

where β denotes the jump in amplitude. This model allows variation (noises) from two consecutive data points to differ by β units, which in turn reduces a small variation in amplitude by requiring a large rise in amplitude. In the case where variations in time and amplitude are allowed, the general reduced function can be set as:

(6) $$y_{\left(\alpha \cdot i-1\right)}=\{\begin{array}{c} 0\;,\;x_{\left(\alpha \cdot i+1\right)}\;\leq \beta +\;x_{\left(\alpha \cdot i-1\right)}\\ 1\;,\;\;x_{\left(\alpha \cdot i+1\right)}\;>\beta +\;x_{\left(\alpha \cdot i-1\right)} \end{array}$$

A proper choice of  β may reduce impacts from noises and a suitable scaling factor α could result in an ideal reduced binary pattern. Therefore, appropriate tuning of either parameter will improve the verification accuracy.

Table 1 and Algorithm 1 include notations and the main pseudo code of the advanced RBP algorithm. The main pseudo code consists of two code segments: an ECG datum x is converted to the statistical counter CT and the rank values are sorted to obtain RK. To expedite our computation process, an unsigned  σ of length  m is created and each bit of σ stores the corresponding value of $b_{k}=\left\{y_{k+1},\;\;y_{k+2},\ldots ,y_{k+m-1}\right\}$. We also set  CT, of length $2^{m}$, to accumulate the repeated number of m-bit words in the reduced binary pattern.

**Table 1:** Notation for the advanced RBP (Reduced Binary Pattern) pseudo code.

Symbols	Description
*CT*	*m*-bit word counter of size 2^m^ for the reduced binary patterns
*N*	Maximum length of the ECG segment for verification
*p*	Relative frequency array
*RK*	Rank array
*m*	Number of bits in a word for the reduced binary pattern
*x*	Input ECG data
*α*	Scaling factor of interval for the reduced binary sequence
*β*	Granularity of amplitude for reduced binary function
*θ_CT_*	The total number of *m*-bit words for counting
*σ*	Unsigned integer with *m*-bits for the purpose of shifting
*τ*	Position variable

**Algorithm 1** Main Pseudo Code of the Advanced RBP Algorithm1: LOOP i UNTIL N BY α2:   σ=13:   IF $x_{\alpha \cdot i+1}$-$x_{\alpha \cdot i-1}$ > β4:    σ++5:   IF i≥m 6:    $\theta_{CT}$++7:    $CT_{\sigma }++$8: LOOP j UNTIL $\theta_{CT}$9:  τ=110:   LOOP k UNTIL $\theta_{CT}$11:     IF j<k12:      IF $CT_{j}\leq \;CT_{k}$13:       τ++

### 4.3. The Evolving RBP Algorithm

Since ECG signals change slightly day by day, modifying our algorithm to handle this issue seems crucial. This model utilizes an incremental learning process to improve the advanced version. The advanced RBP algorithm evolves an incremental-update mechanism for the rank order of the m-bit word $\;j=w_{k}$. If both ECG signals, the obtained one and the original one, come from the same individual, the identity match passes. The original relative frequency p(j) is now replaced by the new relative frequency $p_{new}\left(j\right)$ from the new input ECG.

(7) $$p\left(j\right)=\left(1-\gamma \right)\cdot p\left(j\right)+\gamma \cdot p_{new}\left(j\right)$$

where γ is the weighted factor controlling the degree of impact from the new frequency $p_{new}\left(j\right)$, $j=0,1,\cdots ,2^{m}-1$. The value of γ is affected by the degree of non-stationarity in the old and new ECG signals. A larger γ indicates that the new data are more non-stationary and the rank R(j), $j=0,1,\cdots ,2^{m}-1$ will then be recalculated and updated. This non-stationary behavior can be modeled by verifying the cross-correlation between these two ECG signals in the future.

## 5. Evaluation and Discussion

### 5.1. Compared Algorithms

Next, we will compare the proposed algorithm with two other schemes from the same feature extraction category. It is noted that both selected waveform-based and wavelet-based algorithms require R-R detection and noise preprocessing, which can be totally bypassed in our model.

**Figure 9:**
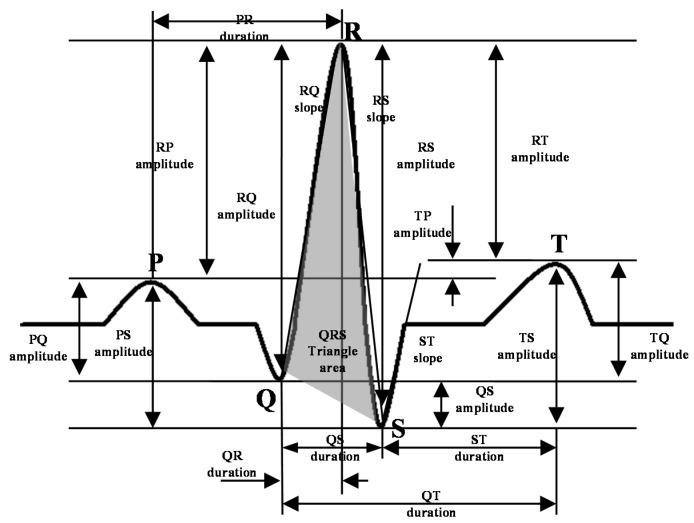
Waveform feature extraction for verification.

In a waveform-based study [13], a total of 19 features are extracted from the four classes: amplitude (PQ, RQ, TQ, RT, PS, RP, TS, RS, PT, QS), duration (QS, PR, QR, ST, QT), slope (RS, ST, and QR), and area (area of the QRS triangle). Descriptions of these features are presented in Figure 9. These features form a feature vector S. The closeness between two feature vectors ${\text{S}}_{1}$ and ${\text{S}}_{2}$ is considered as their distance $d\left({\text{S}}_{1},{\text{S}}_{2}\right)$; the intra- and inter-group distances can be evaluated through Equation (3).

The procedures of the wavelet-based algorithm [12] in comparison include the following: each R-R cardiac cycle is obtained through R-R detection; an interpolation is performed on the R-R interval so each R-R cardiac cycle holds 284 data points; every R-R cycle is cut into three parts, each containing 85, 156, and 43 points; the first 85 and the last 43 points in each R-R cycle are assembled to form a 128-point segment; every four segments are grouped and an n-level discrete wavelet transform (DWT) is performed to obtain the corresponding wavelet coefficients. Four of the computed wavelet coefficients are gathered as a wavelet vector and expressed as:

(8) $$\text{S}=\left[a_{n},d_{n},d_{n-1},d_{1}\right]$$

**Figure 10:**
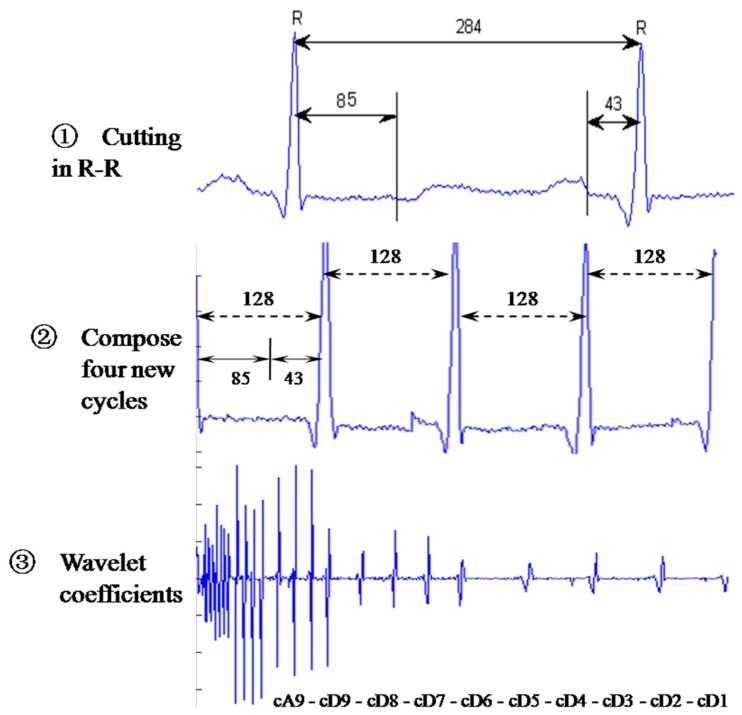
Procedure of the wavelet-based algorithm.

The Euclidean distance between two wavelet vectors ${\text{S}}_{1}$ and ${\text{S}}_{2}$ is regarded as their distance $d\left({\text{S}}_{1},{\text{S}}_{2}\right)$; the intra- and inter-group distances can then be calculated through Equation (3). An example with n=9 is illustrated in Figure 10.

### 5.2. ECG Database

We conducted a comprehensive experiment on three public ECG databases: the MIT-BIH Arrhythmia Database, the MIT-BIH Normal Sinus Rhythm Database, and a Long-Term Database. Descriptions of these three databases are given below.

1. MIT-BIH Normal Sinus Rhythm Database [34]: This database contains 18 long-term ECG recordings from five men aged 26 to 45 and 13 women aged 20 to 50 who have no significant arrhythmias. These ECG signals were sampled at a rate of 128 Hz and represented by a 12-bit binary sequence.
2. MIT-BIH Arrhythmia Database [35,36]: The database includes 48 groups, each comprising a half-hour two-lead ECG recording, giving a total of 24 hours of information. The data contains 47 individuals’ ECG information (dataset IDs 201 and 202 are duplicated); subjects consist of 25 men aged between 32 and 89 and 22 women aged from 23 to 89. These ECG data were sampled at 360 Hz and use a 12-bit binary storage.
3. Long-Term Database [33]: The evolving RBP algorithm was tested on a database consisting of different segments of ECG signals collected for up to six months. These 310 ECG recordings, containing a 20-second Lead-I signal digitized at 500 Hz and a 12-bit binary expression, are obtained from 44 men and 46 women aged 13 to 75. The number of recordings for each person varies from two to 20.

### 5.3. Measurement Approaches

Two approaches are used to evaluate our implemented algorithms:

1. Success rate: This is a metric used for accuracy measurement. Based on the results of comparisons between the individuals, when the inter-subject distance is smaller than the average inter-subject distance, we considered it an identification error. Summing up these errors gives us the total number of errors; then we divided this figure by the total number of comparisons to give the success rate.
2. False Acceptance (FA) and False Rejection (FR) rates: These are also the metrics used for accuracy performance. The FR denotes the relative ratio of subjects that should be accepted but are actually rejected by the classifier; similarly, the FA is the ratio of subjects that should be rejected but are actually accepted by the classifier. The threshold for FA/FR is obtained from the training set, which aims to minimize $\frac{FA+FR}{2}$.

### 5.4. Experimental Results

#### 5.4.1. Basic RBP Algorithm

To verify how efficient this algorithm is in human verification by ECG, two types of comparisons are considered: self- and subject-comparison.

1. Self-comparison: Two eight-segment data are arbitrarily selected from one individual and their corresponding distances are measured using Equation (2). Each segment contains 3600 data points (10 s). All 64 intra-subject distances obtained from segments 1 to 8 for the subject ID number 100 in the MIT-BIH Arrhythmia Database are listed in Table 2. It is noted that all entries are symmetric with diagonal entries being zero since they denote the distances between two identical segments.

**Table 2:** Self-comparison for subject ID 100.

	1	2	3	4	5	6	7	8
1	0	0.033	0.033	0.035	0.040	0.037	0.036	0.033
2	0.033	0	0.028	0.039	0.047	0.045	0.036	0.045
3	0.033	0.028	0	0.033	0.042	0.042	0.031	0.039
4	0.035	0.039	0.033	0	0.035	0.041	0.030	0.034
5	0.040	0.047	0.042	0.035	0	0.032	0.037	0.023
6	0.037	0.045	0.042	0.041	0.032	0	0.043	0.033
7	0.036	0.036	0.031	0.030	0.037	0.043	0	0.034
8	0.033	0.045	0.039	0.034	0.023	0.033	0.034	0

2 Subject-comparison: Within the same database, two eight-segment data are chosen from two distinct individuals, one from each, and the distance between each pair of subjects is evaluated. The results of all 64 inter-subject distances for the pair of subjects with ID numbers 100 and 101 from the MIT-BIH arrhythmia database are shown in Table 3.

**Table 3:** Subject-comparison between subject IDs 100 and 101.

	1	2	3	4	5	6	7	8
1	0.045	0.040	0.051	0.057	0.047	0.046	0.045	0.044
2	0.051	0.041	0.055	0.055	0.052	0.048	0.049	0.052
3	0.052	0.041	0.056	0.060	0.052	0.046	0.043	0.048
4	0.049	0.045	0.051	0.054	0.050	0.053	0.037	0.047
5	0.043	0.041	0.038	0.060	0.037	0.045	0.037	0.042
6	0.052	0.039	0.052	0.065	0.046	0.048	0.045	0.040
7	0.052	0.046	0.057	0.056	0.054	0.055	0.044	0.052
8	0.043	0.041	0.043	0.060	0.039	0.047	0.037	0.041

Next, we measure the average distance between two subjects from the same database using Equation (3). Table 4 lists all 64 average intra-group distances for subject IDs 100 to 107 from the MIT-BIH Arrhythmia Database. For example, the first row and first column record the average of all entries in Table 2; the average of all values in Table 3 is listed in the first row and second column and in the second row and first column.

**Table 4:** Basic RBP (Reduced Binary Pattern) comparison result for eight subject IDs: 100–107.

	100	101	102	103	104	105	106	107
100	0.032	0.048	0.045	0.061	0.058	0.059	0.056	0.062
101	0.048	0.028	0.040	0.044	0.041	0.036	0.039	0.031
102	0.045	0.040	0.031	0.048	0.044	0.042	0.048	0.032
103	0.061	0.044	0.048	0.016	0.034	0.030	0.043	0.009
104	0.058	0.041	0.044	0.034	0.029	0.029	0.039	0.013
105	0.059	0.036	0.042	0.030	0.029	0.017	0.031	0.008
106	0.056	0.039	0.048	0.043	0.039	0.031	0.027	0.022
107	0.062	0.031	0.032	0.009	0.013	0.008	0.022	0.001

Intuition suggests that a strong association should exist between certain patterns with each individual; therefore, the intra-subject distances must be smaller than the intra-group distances. Thus, it makes sense to treat the opposite cases as verification errors. Similarly, the RBP algorithm is applied to subjects in the MIT-BIH normal sinus rhythm database as well. The experimental results show that the success rates for the two groups of people, with and without significant arrhythmias, are 95.791% and 90.196%, respectively.

To seek better accuracy, the effect of the length of the m-bit word is considered. Using 10 s periods as the duration of the input data should be reasonable for a fair evaluation. A self-comparison experiment is conducted to examine whether the value of  m and the stability of identity detection are related. Two 31-segment data, each containing a 10 s period, are selected from subject ID 100 in the MIT-BIH Arrhythmia Database. The results of all intra-subject distances obtained using Equation (3) are measured for different values of  m, as shown in Table 5. It is clear that the distances vary abruptly when m = 4 and become more stable as m increases. However, a bigger m leads to computational and space complications. To balance the trade-offs, we decide to set m=8 in this study.

**Table 5:** Mean and standard deviation of 30 distances for each m.

	Intra-Subject Distances for Each m							
m	4	5	6	7	8	9	10	11
Mean	0.0273	0.0355	0.0444	0.0373	0.0361	0.0345	0.0322	0.0292
Standard deviation	0.0173	0.0110	0.0104	0.0045	0.0036	0.0028	0.0025	0.0023

#### 5.4.2. Advanced RBP Algorithm

Two parameters, interval and amplitude, are considered in this advanced design. Therefore, the pair of data points $x_{\left(\alpha \cdot i+1\right)}$ and $x_{\left(\alpha \cdot i-1\right)}$, instead of $x_{i+1}$ and $x_{i}$, are compared to obtain the reduced binary pattern via Equation (4), and the ECG data are examined not only locally but globally.

**Figure 11:**
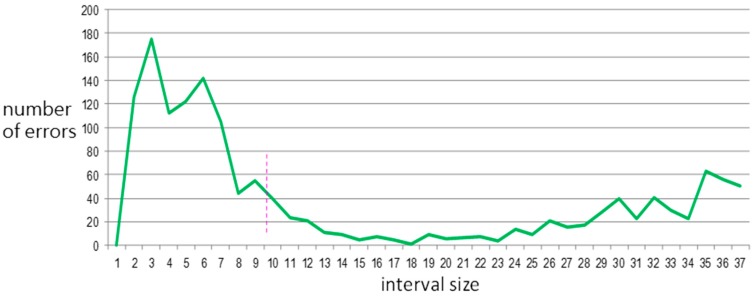
Total number of errors *versus* interval size for Arrhythmia Database.

**Figure 12:**
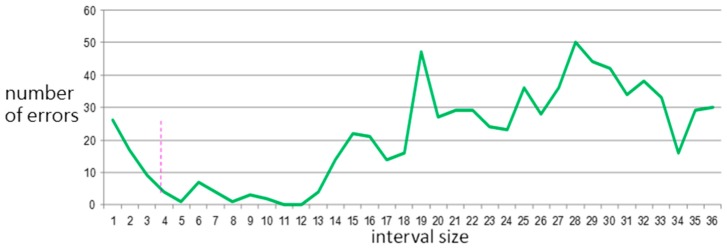
Total number of errors *versus* interval size for Normal Database.

Experiments with 1 to 36 intervals were conducted; Figure 11 and Figure 12 show their effects on the total number of verification errors in the two databases.

The total number of errors shows a sharp drop followed by a short stable zone for α=15 in the Arrhythmia Database and for α=5  in the Normal Database. Since these two databases are sampled at 360 Hz and 128 Hz, respectively, α=15 and α=5 correspond to down-sample signals at 24 Hz and 25.6 Hz, respectively. These frequencies are quite close to the 25 Hz bandwidth. This new finding is analogous to the bandwidth of digitized ECG data in [4]. If a signal is sampled at a higher or lower rate, it may cause unnecessary noise or provide insufficient information, respectively. Therefore, 25 Hz seems to be a suitable sampling rate.

In addition to interval tuning, an experiment using the MIT-BIH Arrhythmia Database was conducted to check how recognition accuracy changes when the amplitude is adjusted. Table 6 and Table 7 record the results of both algorithms with α=10. The data show that the success rate in the advanced model is a lot better than that in the basic model. When the increase in amplitude β=1, the impact of the signal noise is reduced to give the results showing the best performance; when β>1, certain distinguishing features may be removed for verification and result in a lower success rate. β of different amplitudes using basic RBP algorithm with α=10.

**Table 6:** Performance for β of different amplitudes using advanced RBP algorithm with α=10.

Amplitude β	1	2	3	4	5	10	15	20
Total number of errors	71	256	329	396	437	410	388	422
Success rate	0.967	0.881	0.847	0.816	0.797	0.810	0.820	0.804

**Table 7:** Performance for β of different amplitudes using advanced RBP algorithm with α=10.

Amplitude β	1	2	3	4	5
Total number of errors	29	33	73	159	222
Success rate	0.987	0.985	0.966	0.926	0.897

The evaluation of our algorithms, the basic and the advanced RBP with α=5 an β=1, will depend on comparisons with two other feature extraction algorithms: a waveform-based algorithm with 19 waveform features extracted [13] and a transform-based scheme with wavelet feature extraction [12]. It is worth mentioning that R-R detection and noise preprocessing are required in both of the other algorithms but can be completely bypassed in ours.

In the evaluation using the MIT-BIH Arrhythmia and Normal databases, it is obvious from the comparison of outcomes shown in Table 8 that the waveform-based algorithm with 19 features performs well, but our advanced RBP algorithm still excels, having an extremely high success rate in both public databases.

**Table 8:** Total number of errors and success rate for comparison.

Algorithm	Arrhythmia		Normal	
	Total Number of Errors	Success Rate	Total Number of Errors	Success Rate
Waveform	14	99.352%	1	99.673%
Wavelet Transform	14	98.242%	19	93.791%
Basic RBP	97	95.513%	30	92.484%
Advanced RBP	7	99.676%	0	100.000%

The FA and FR ratios for the normal sinus rhythm and arrhythmia databases are listed in Table 9. Here the associated parameters for the normal sinus rhythm database are m=8 and α=5 with a sampling rate of 128 Hz and m=8 and α=15 with a sampling rate of 360 Hz. For the purpose of personal verification, the false rejection and acceptance rates should be as small as possible. The advanced RBP algorithm has been tested 18 × 8 = 144 times and 47 × 3 = 147 times for Normal and Arrhythmia databases, respectively, and advanced RBP has a false rejection rate of around 1.67% and a false acceptance rate of 1.43% for the Normal Database. Thus the performance of our algorithm should be acceptable.

**Table 9:** False rejection and acceptance ratios of advanced RBP algorithm.

MIT-BIH Database	FR	FA
Normal	0.016748	0.014319
Arrhythmia	0.068918	0.059783
Average	0.042833	0.037051

Table 10 provides the information on execution time for all algorithms under comparison. It is clear that the execution time for the advanced scheme is shorter than that for the basic RBP algorithm, whose performance is definitely at least nine times faster than those of the waveform- and transform-based algorithms.

**Table 10:** Execution times of the compared algorithms.

Algorithms	Execution Time Per Cycle
Waveform	0.113675 s
Wavelet	0.225854 s
Basic RBP	0.013305 s
Advanced RBP	0.008565 s

#### 5.4.3. Evolving RBP Algorithm

The performance of the evolving RBP algorithm is evaluated on the long-term ECG database, where 20 individuals’ ECG data recorded over 54 days were selected. All subjects had their ECGs measured on a minimum of two and a maximum of six days. Table 11 contains the data for the subject ID person_01. In this study, the evolving RBP algorithm, tested on the long-term database, is implemented with α=13 and β=1  in the advanced RBP model.

**Table 11:** Records of distribution for person_01 on five days.

Date	Record Number
2004-12-07	1, 2
2004-12-28	3, 4, 5, 6, 7, 8, 9
2005-03-15	10, 11, 12, 13
2005-04-05	14, 15, 16, 17
2005-04-26	18, 19, 20

**Figure 13:**
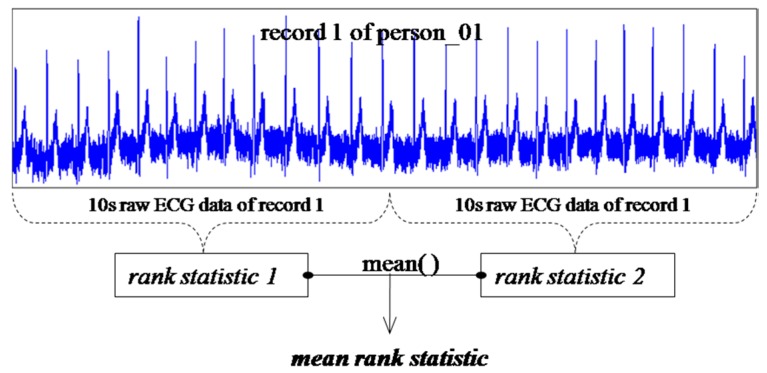
Mean rank statistic for a new ECG signal.

Not all subjects have six days’ worth of ECG data; for most of them, only two or three days’ worth of data is on record. Each recorded ECG is 20 s long and is cut into two equal-length segments of 10 s each. Each segment is sorted into rank statistics after reduced binary conversion and m-bit word counting and, finally, we get the mean rank statistic $RK_{mean}$ of rank statistics $RK_{1}$ and $RK_{2}$ for the two segments, respectively. This approach is illustrated in Figure 13. Table 12 gives us the result for person_01 by applying the non-evolving advanced RBP approach to calculate the similarity distance using Equation (2) between the first record (record No. 1) and other records.

**Table 12:** Similarity distance of non-evolving RBP for person_01.

Date	Records						
2004-12-07	0.079						
2004-12-28	0.120	0.093	0.104	0.102	0.092	0.117	0.226
2005-03-15	0.103	0.100	0.105	0.103			
2005-04-05	0.100	0.096	0.098	0.112			
2005-04-26	0.101	0.094	0.071				

The evolving RBP scheme Equation (6) models the rank statistics on the average relative frequency array of the first record on the first day from the same individual, and follows with an update of the first record on another day. The weight distribution between the model and the new record is 0.5:0.5. To be precise, each day, we simply use the first valid record to update the model, while the other record serves for comparison purposes only and is not used for the update. The similarity distance between the model and recorded data is shown in Table 13, where cells with grey backgrounds indicate that the corresponding recorded data are updated into the model. Comparing these results with those presented in Table 11, it can be seen that the evolving RBP approach has a much smaller similarity distance than the advanced RBP one.

**Table 13:** Similarity distance when applying the evolving RBP to person_01.

Date	Records						
2004-12-07	0.079						
2004-12-28	0.119	0.047	0.055	0.044	0.049	0.071	0.201
2005-03-15	0.049	0.227	0.0174	0.019			
2005-04-05	0.015	0.012	0.018	0.017			
2005-04-26	0.012	0.012	0.018				

In order to obtain a whole valid evaluation between the evolving and advanced RBP algorithms, we sum all the similarity distances after the update points on each date as *S_arbp_* and *S_erbp_* for the advanced RBP algorithm and the evolving RBP algorithm, respectively, which are the non-grey records in the second-to-fifth rows in Table 13. Their values are shown in Table 14, which demonstrates an improved rate of evolving RBP to advanced RBP is 26.47%, which is obtained by (*S_arbp_* − *S_erbp_*)/*S_arbp_*, confirming that the evolving process is effective. This result reveals that the evolving process of the RBP algorithm does improve the verification performance for the non-stationary behavior in long-term ECG signals.

**Table 14:** Improved rate of the evolving process.

S_arbp_	S_erbp_	Improved Rate
4.0516	2.9790	26.47%

As shown in Table 15, we use the same database and a procedure similar to the similarity distances evaluation to measure the false acceptance (FA) and false rejection (FR) rates for the evolving RBP, and the result shows that evolving RBP has improved rates of 43.77% and 9.57% for FA and FR, respectively. The average improved rate is 25.25%, which is quite close to the improvement of the success rate, which was 26.47%.

**Table 15:** FA (false acceptance)/FR (false rejection) of the evolving process.

Item	Advanced RBP	Evolving RBP	Improved Rate
FA	0.3556	0.1999	0.4377
FR	0.4200	0.3797	0.0957
(FA+FR)/2	0.3878	0.2898	0.2525

## 6. Conclusions

In this paper, a novel ECG card architecture and algorithm for ECG human verification are proposed. Verifications tested on subjects from the two public MIT-BIH databases confirm that the RBP algorithm performs in a timely manner with low computational complexity and is rather efficient in ECG human identity recognition. Moreover, the RBP scheme is enhanced by tuning the parameters, interval, and amplitude between sample points. The advanced RBP design demonstrates good accuracy with much shorter execution duration than those of the waveform- and transform-based algorithms. Furthermore, the modified evolving RBP algorithm cannot only easily merge the new rank data into the old one, but it is also capable of handling non-stationary ECG signals.

## Supplementary Materials

Supplementary materials can be accessed at: http://www.mdpi.com/1424-8220/15/8/20730/s1.
