# Supplementary material for: ECG Sensor Card with Evolving RBP Algorithms for Human Verification
Source: Sensors (Basel). 2015 Aug 21;15(8):20730–51. doi: 10.3390/s150820730 (PMC4570445; doi:10.3390/s150820730)
Supplement: Supplementary file 1 [file sensors-15-20730-s001.zip › sensors-15-20730-s001.pdf]

OPEN ACCESS

sensors

ISSN 1424-8220

www.mdpi.com/journal/sensors

Supplementary Information

ECG Sensor Card with Evolving RBP Algorithms for Human Verification. *Sensors* 2015, *15*, 20730–20751

Kuo-Kun Tseng [1,*], Huang-Nan Huang [2,*], Fufu Zeng [1] and Shu-Yi Tu [3]

[1] Department of Computer Science and Technology, Harbin Institute of Technology, Shenzhen Graduate School, Shenzhen 518055, China; E-Mail: 12S051058@hitsz.edu.cn

[2] Department of Mathematics, Tunghai University, Taichung 40704, Taiwan

[3] Department of Mathematics, University of Michigan, Flint 48502, MI, USA; E-Mail: sytu@umflint.edu

* Authors to whom correspondence should be addressed; E-Mails: kktseng@hitsz.edu.cn (K.-K.T); nhuang@thu.edu.tw (H.-N.H); TEL: +86-134-1873-5211 (K.-K.T); FAX: +86-755-2603-3008 (K.-K.T).

---

1. Introduction

This document is a source code description for the paper “ECG Sensor Card with Evolving RBP Algorithms for Human Verification”.

2. Database

The ECG database used in our paper is stored in the “ECGdatabase” file and has three subfiles: “arrhythmia”, “normal”, and Longterm. There are three ECG databases in this package. The original databases can be found as follows, and the complete data sets can be retrieved from the corresponding websites.

(a) MIT-BIH Arrhythmia Database: http://physionet.org/physiobank/database/mitdb/
(b) MIT-BIH Normal Sinus Rhythm Database: http://www.physionet.org/physiobank/database/nsrdb/
(c) Physionet Long-term Database: https://physionet.org/works/BiometricHuman IdentificationbasedonECG

The database is in the EcgDatabase directory (physionet_project directory). It contains the data of 90 persons and has a total of 310 records. The recording dates are written in the corresponding .hea files.

3. Operation

Several steps are used to perform this evaluation.

a) Success Rate Comparison

- The two whole databases, the MIT-BIH Arrhythmia and Normal databases, should be downloaded from the original website.
- Open one MATLAB source code file, such as “waveform.m”, and modify the variance “path” in the header of the file with the full path of the subfile (“arrhythmia” or “normal” file) of the “ECGdatabase”, such as “C:\code\ECGdatabase\arrhythmia”.
- Finally, click the “run” button to obtain the result. The cross-validation takes some time, especially for the arrhythmia database, which has 47 individuals.

b) FA/FR of ARBP and Euclidean Distance

- The two whole databases, the MIT-BIH Arrhythmia and Normal databases, should be downloaded from the original website.
- Then, open and click the “run” button for advancedRBP_FRFA.m and euclidean_FRFA.m to obtain the results.

c) Evolving RBP FA/FA

- The Long-term Physionet Database should be downloaded from the original website.
- Open and click the “run” button for noEvolvingRBP.m and evolvingRBP.m to obtain the results of the comparison.

4. Source Code

d) Success Rate Comparison

advancedRBP.m, basicRBP.m, waveform.m, and waveletTransform.m are the corresponding algorithms for the comparison. They all work with the same two databases for success rate comparison.

e) FA/FR of ARBP

Moreover, for further information about advanced RBP, advancedRBP_FRFA.m and euclidean_FRFA.m are provided for the purpose of comparing advanced RBP and Euclidean distance with regard to false rejection and acceptance rates.

f) Evolving RBP FA/FA

- noEvolvingRBP.m: computes the FR (false rejection rate) and FA (false acceptance rate) of the advanced RBP algorithm under the no-incremental-update condition;
- evolvingRBP.m: calculates the FR (false rejection rate) and FA (false acceptance rate) of the advanced RBP algorithm under the fresh condition;
- similar.m, getDatPath, and rankStatistic.m are the ancillary files used in the evaluation.

5. Output Analysis

a) Success Rate Comparison

The results can be seen in the "workspace" of MATLAB directly. The detailed results are stored under the variable "*meanDm*", which has two dimensions: the first denotes the reference person and the second the compared person. The statistical information of the correct rate is stored under the variable "*statistic*" in the workspace, or the output result can also be stored in an Excel data file. The store position is "C:\code", and they are named in the form "source code filename-ECG database name", for example, "waveform-arrhythmia.xlsx" and "waveform-normal.xlsx".

The Excel results are illustrated for 18 individuals below using "waveform-normal.xlsx" as an example.

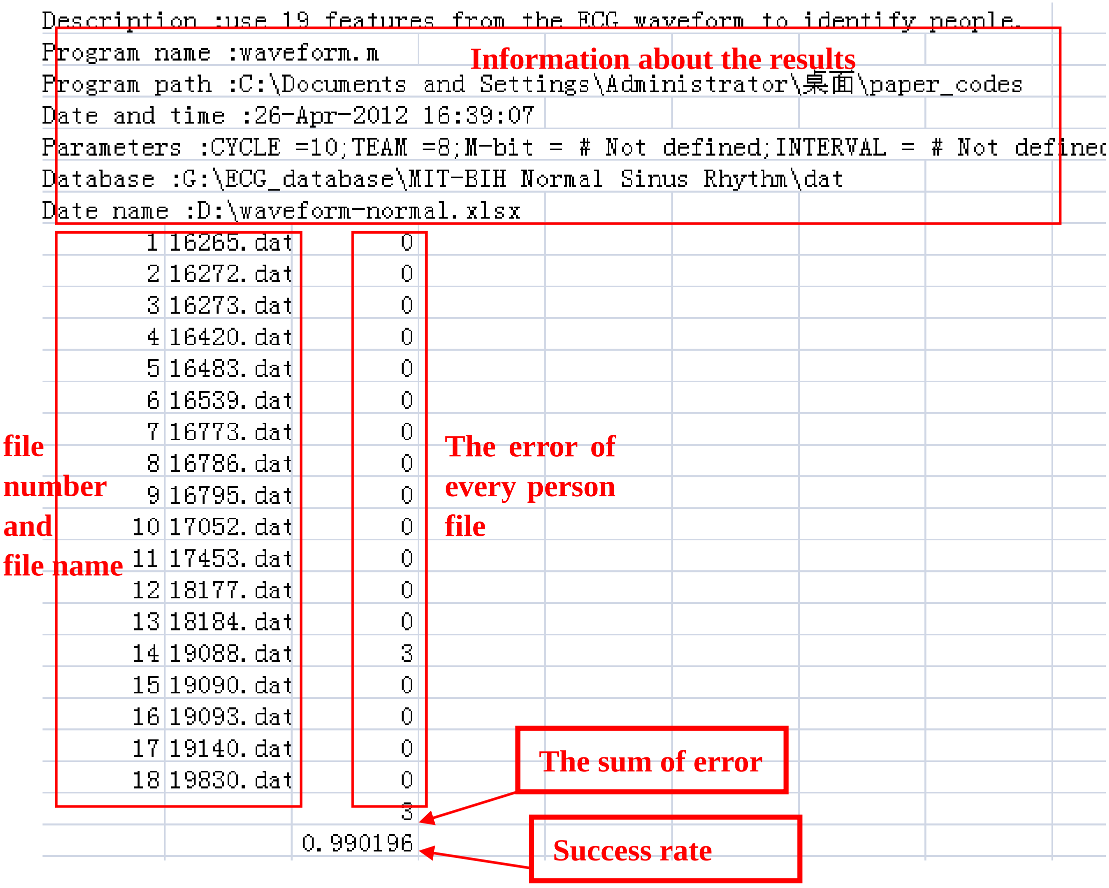

Description :use 19 features from the ECG waveform to identify people.
Program name :waveform.m
Program path :C:\Documents and Settings\Administrator\桌面\paper_codes
Date and time :26-Apr-2012 16:39:07
Parameters :CYCLE =10;TEAM =8;M-bit = # Not defined;INTERVAL = # Not define
Database :G:\ECG_database\MIT-BIH Normal Sinus Rhythm\dat
Date name :D:\waveform-normal.xlsx

1	16265.dat	0
2	16272.dat	0
3	16273.dat	0
4	16420.dat	0
5	16483.dat	0
6	16539.dat	0
7	16773.dat	0
8	16786.dat	0
9	16795.dat	0
10	17052.dat	0
11	17453.dat	0
12	18177.dat	0
13	18184.dat	0
14	19088.dat	3
15	19090.dat	0
16	19093.dat	0
17	19140.dat	0
18	19830.dat	0
		3
	0.990196	

Figure S1. The results of success rate for 18 individuals.

The above data is from Sheet 1 of "waveform-normal.xlsx", and there is a detailed result on Sheet 2 of "waveform-normal.xlsx".

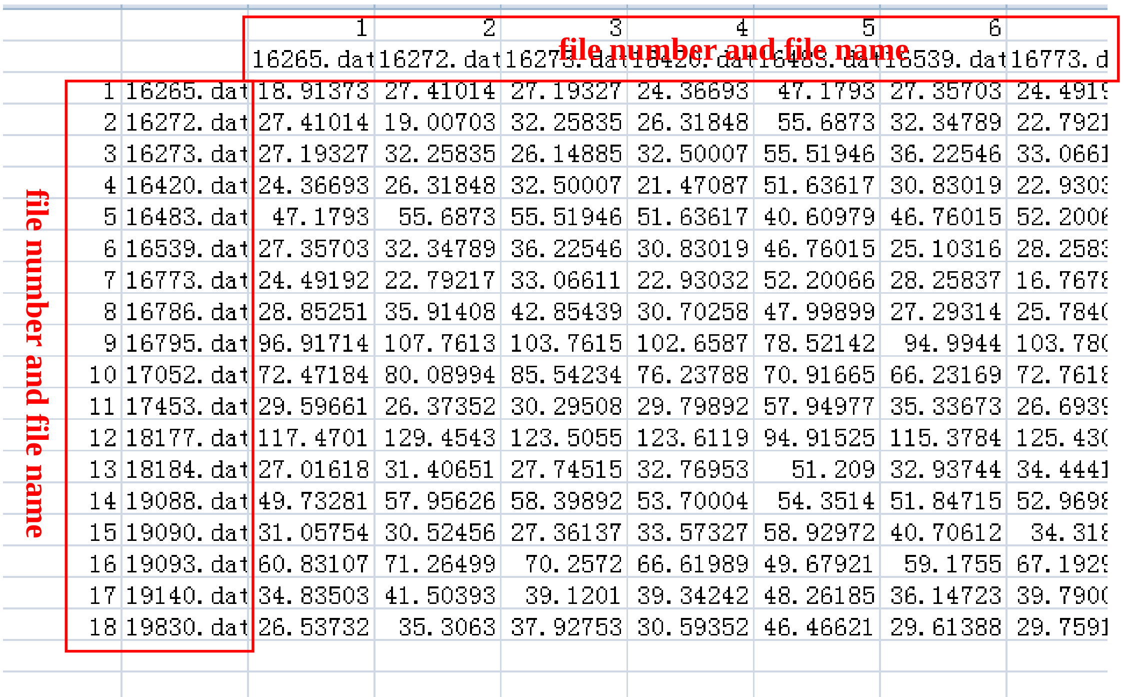

		1	2	3	4	5	6	
		16265.dat	16272.dat	16273.dat	16420.dat	16483.dat	16539.dat	16773.d
1	16265.dat	18.91373	27.41014	27.19327	24.36693	47.1793	27.35703	24.491
2	16272.dat	27.41014	19.00703	32.25835	26.31848	55.6873	32.34789	22.7921
3	16273.dat	27.19327	32.25835	26.14885	32.50007	55.51946	36.22546	33.0661
4	16420.dat	24.36693	26.31848	32.50007	21.47087	51.63617	30.83019	22.930
5	16483.dat	47.1793	55.6873	55.51946	51.63617	40.60979	46.76015	52.200
6	16539.dat	27.35703	32.34789	36.22546	30.83019	46.76015	25.10316	28.258
7	16773.dat	24.49192	22.79217	33.06611	22.93032	52.20066	28.25837	16.767
8	16786.dat	28.85251	35.91408	42.85439	30.70258	47.99899	27.29314	25.784
9	16795.dat	96.91714	107.7613	103.7615	102.6587	78.52142	94.9944	103.78
10	17052.dat	72.47184	80.08994	85.54234	76.23788	70.91665	66.23169	72.761
11	17453.dat	29.59661	26.37352	30.29508	29.79892	57.94977	35.33673	26.693
12	18177.dat	117.4701	129.4543	123.5055	123.6119	94.91525	115.3784	125.43
13	18184.dat	27.01618	31.40651	27.74515	32.76953	51.209	32.93744	34.4441
14	19088.dat	49.73281	57.95626	58.39892	53.70004	54.3514	51.84715	52.969
15	19090.dat	31.05754	30.52456	27.36137	33.57327	58.92972	40.70612	34.31
16	19093.dat	60.83107	71.26499	70.2572	66.61989	49.67921	59.1755	67.192
17	19140.dat	34.83503	41.50393	39.1201	39.34242	48.26185	36.14723	39.790
18	19830.dat	26.53732	35.3063	37.92753	30.59352	46.46621	29.61388	29.7591

Figure S2. The detailed result of success rate comparison for 18 individuals.

b) FA/FR Results of ARBP and Evolving RBP FA/FA

The overall results are shown in the command line window, as follows:

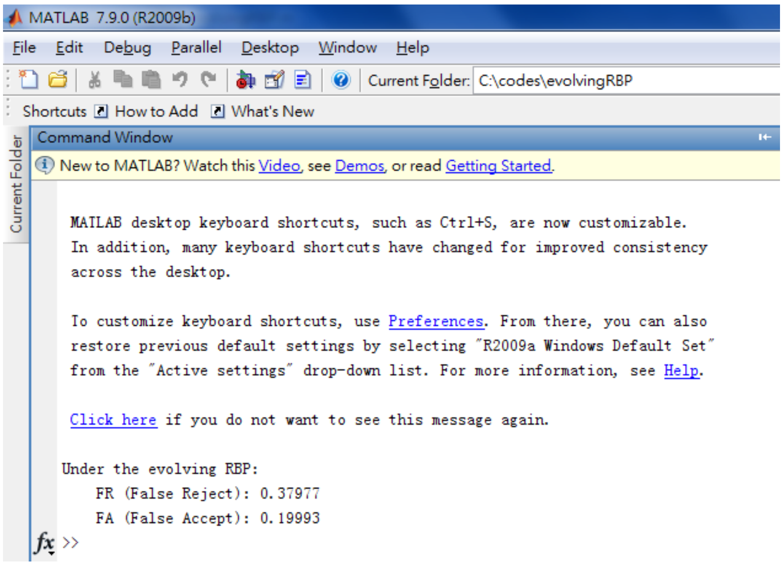

Figure S3. The overall result on Matlab.

© 2015 by the authors; licensee MDPI, Basel, Switzerland. This article is an open access article distributed under the terms and conditions of the Creative Commons Attribution license (http://creativecommons.org/licenses/by/4.0/).
