# Supplementary material for: ECG Sensor Card with Evolving RBP Algorithms for Human Verification
Source: Sensors (Basel). 2015 Aug 21;15(8):20730–51. doi: 10.3390/s150820730 (PMC4570445; doi:10.3390/s150820730)
Supplement: Supplementary file 1 [file sensors-15-20730-s001.zip › codes_for_sensors/codes/evaluation/Instruction.docx]

In the evaluation directory, it contains:

***noEvolvingRBP.m***: calculate the FR (false reject) and FA (false accept) of the advanced RBP algorithm under the no fresh condition;

***evolvingRBP.m***: calculate the FR (false reject) and FA (false accept) of the advanced RBP algorithm under the fresh condition;

*similar.m*, *getDatPath* and *rankStatistic.m* are the ancillary files of evaluation.

Its database is in the EcgDatabase directory (physionet_project directory), it contains 90 persons, having 310 records totally, the recording date is written in the corresponding .hea files.

This evaluation is designed for the long time ECG identification with advanced RBP algorithm.
